# Laying a Strong Foundation with a Novel “Basal-Bolus” Point of Care Ultrasound Curriculum for Internal Medicine Residents

**DOI:** 10.24908/pocusj.v11i01.20051

**Published:** 2026-04-22

**Authors:** Erin M. Finn, John R. Stephens, Hillary Spangler, Margaret Fennell, Olivia Hardy, Ria Dancel

**Affiliations:** 1 Departments of Internal Medicine and Pediatrics, Division of Hospital Medicine, University of North Carolina, Chapel Hill, NC, USA; 2 Department of Medicine, Division of Geriatric Medicine, University of North Carolina, Chapel Hill, NC, USA; 3 Internal Medicine and Pediatrics Residency Program, University of North Carolina, Chapel Hill, NC, USA

**Keywords:** POCUS, Graduate Medical Education, Internal Medicine, Point of Care Ultrasound

## Abstract

**Background:**

While professional societies acknowledge the importance of point of care ultrasound (POCUS), a minority of internal medicine (IM) residencies have a formal curriculum. This study aims to 1) describe the implementation of a longitudinal POCUS curriculum for first-year IM trainees and 2) assess the effectiveness of the curriculum in improving knowledge and confidence.

**Methods:**

We implemented a longitudinal curriculum for IM interns, with didactic and hands-on sessions throughout the year. We assessed curriculum effectiveness through knowledge tests and confidence surveys at the beginning and end of the intern year. Trainees who took our POCUS elective were given the same tests to assess knowledge retention.

**Results:**

Between 2021 and 2024, 87 interns completed the curriculum; 37 (42.5%) completed pre- and post-test questionnaires. Mean scores for knowledge tests improved from 44.4% to 62.9% (mean difference 18.5%, 95% CI 14.9-22.1, p < 0.001). Confidence in using POCUS to identify pathologic findings and apply it in clinical scenarios improved for all 17 measures (p < 0.001). Of the 37 interns who completed the knowledge assessments, 15 (40.5%) took the upper-level elective and completed knowledge assessments. There were no differences between first-year post-curriculum and elective pre-curriculum scores.

**Conclusions:**

Knowledge scores and confidence in POCUS improved following a longitudinal curriculum for internal medicine interns. Residents participating in a subsequent POCUS elective maintained their knowledge scores.

## Introduction

Point of care ultrasound (POCUS) is defined as an ultrasound exam performed at the bedside by a healthcare provider to answer a diagnostic question or to guide an invasive procedure [1]. There is much evidence that POCUS improves the safety and success of procedures, expedites and improves diagnosis and treatment, and improves patient satisfaction [2–8]. Both the Alliance for Academic Internal Medicine (AAIM) and the American College of Physicians (ACP) have formally acknowledged the important role of POCUS in internal medicine (IM) and support the integration of POCUS into graduate medical education [9,10].

LoPresti et al. reviewed the current use of POCUS among IM residency programs using the 2020 Association of Program Directors in Internal Medicine (APDIM) program directors' survey [11]. They found that of the existing United States IM residency programs that responded to the survey, greater than 95% reported that their residents are exposed to diagnostic POCUS, but only 35% provide formal teaching to all residents. While POCUS curricula have been described for trainees in a number of disciplines, relatively few published studies have described curricula within the field of IM [12–19].

At our institution, we have developed the “Basal-Bolus POCUS Curriculum,” which consists of a mandatory curriculum that spans the first year of training (the “basal” component), which can be followed by intensive 2-week electives in diagnostic and procedural POCUS offered to second- through fourth-year residents (the “bolus” components). We describe the implementation and outcomes of our longitudinal IM curriculum for first-year residents (“interns”).

## Methods

### Setting and participants

Our longitudinal intern POCUS curriculum was implemented at the University of North Carolina IM Residency program at the beginning of the academic year 2021. The residency program has 95–98 residents total, with 29–30 interns per year. Every categorical intern participates in the curriculum.

### Study design

We performed a retrospective analysis of curriculum implementation, comparing pre- and post-curriculum measures. This study was exempted from review by the Institutional Review Board of the University of North Carolina (IRB 19-1013).

### Curriculum development

**Figure 1.**
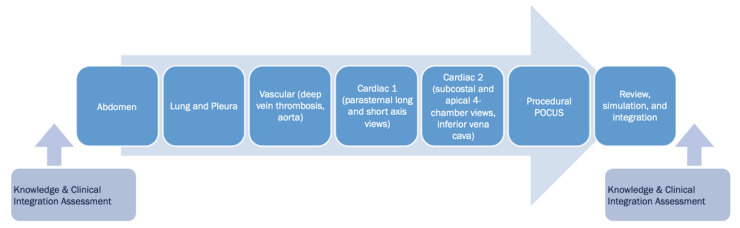
Structure of longitudinal first-year curriculum.

The longitudinal curriculum spanned the academic year, and interns participated in 7 sessions (Figure 1) focusing on abdominal (liver, spleen, kidneys, bladder, deep pelvis), pleural/lung, vascular (deep vein and aorta), cardiac 1 (parasternal long and short axis views), cardiac 2 (apical and subcostal 4 chamber views and inferior vena cava), and procedural POCUS (focusing on 2 procedures chosen by the trainees, such as paracentesis, central lines, thoracentesis, knee arthrocentesis, or lumbar punctures). The seventh and final session was a review and integration session, during which interns demonstrated the POCUS exams they would use to assist in the diagnosis and management of simulated patient cases. The session topics were chosen based on high-yield clinical application to internal medicine, informed by the clinical and teaching experiences of the faculty. Each distinct teaching session was taught three times to accommodate three intern cohorts, which follow an X+Y model, and allowed an optimal instructor-to-learner ratio [20].

Each teaching session lasted 2.5 hours and began with 15–30 minutes of didactic instruction focused on sonographic anatomy. This was followed by 1 hour of hands-on practice and 1 hour of abnormal image review, where the interns learned to systematically evaluate and interpret ultrasound images.

There were typically 9–10 interns per session. Two course directors were each supported with 15% full-time equivalent (FTE) and about five other POCUS-competent core faculty were incentivized to teach. Upper-level residents who had completed the intern longitudinal curriculum were invited to provide near-peer teaching under faculty supervision. To optimize hands-on learning, we maintained a 3:1 learner-to-instructor ratio. The ultrasound machines used for teaching were cart-based machines (Sonosite XPorte or PX, FUJIFILM Sonosite Inc., Bothell, WA, USA) that were either designated for teaching or borrowed from inpatient units. Interns scanned each other on a voluntary, opt-in basis as they typically have optimal imaging windows and normal anatomy. The course was prefaced with the stipulation that all incidental findings would be referred for comprehensive imaging and further evaluation.

Image review consisted of a slide deck of abnormal ultrasound videos facilitated by one of the course directors. For each image, an intern systematically approached the ultrasound clip to comment on the transducer used, the exam type, the appropriateness of the depth and gain of the image, the organ or organs being imaged, the anatomy, and any notable pathology.

Finally, autonomous learning was encouraged outside of the curriculum in several formats. Residents were provided with course lectures and previously-recorded abnormal image sessions to review and test themselves. Additionally, we used a shared POCUS group on a group messaging application to provide a platform to share Health Insurance Portability and Accountability Act (HIPAA)-compliant POCUS images and videos for real-time feedback from experts and as a venue for POCUS instructors to teach learners by guiding them through clinical cases to reinforce teaching points.

### Outcomes and evaluation

We developed a knowledge assessment tool consisting of 56 multiple-choice questions (Supplementary Material S1) with ultrasound clips or still images accompanying each question. The test was created by one of the authors, RD, who was also one of the experts tasked to create the final knowledge assessment exam for the Society of Hospital Medicine and American College of Chest Physicians (CHEST) national POCUS certificate of completion (COC) [21,22]. Our exam was patterned after the COC exam; a challenging and comprehensive exam designed to be taken at the end of a training program geared towards the development of expertise in POCUS.

The assessment was given at the beginning of the intern year and again at the end of the year. Questions were based on a review of literature and ranged from lower-order thinking skills, such as pitfalls in image acquisition and identification of artifacts and structures, to higher-order skills that required image interpretation and clinical integration. Interns were highly encouraged to complete the assessments but are not penalized for failing to do so. Testing was conducted entirely online using Research Electronic Data Capture® (REDCap, Vanderbilt University, Atlanta, GA, USA) and took about 30 minutes for individuals to complete.

We also administered confidence assessments at the beginning and end of the intern year to evaluate resident confidence in simple identification of 14 POCUS findings (e.g., identifying a pericardial effusion, lung consolidation, ascites) and in clinical integration (e.g., using POCUS to evaluate the cause of hypotension, hypoxia, or acute kidney injury). Residents were asked to rate their confidence on a scale of 0 to 100 (Supplementary Material S2). After completing the curriculum, residents were surveyed regarding their attitudes toward POCUS (e.g., how much they agree or disagree with the statement that POCUS adds time to their clinical work or whether it adds value to bedside diagnosis), whether they anticipate continuing to utilize POCUS, and the barriers that might prevent this (Supplementary Material S3). These questions were novel at the time of survey creation but were considered important balancing measures. The tool was reviewed by a statistician with experience in survey creation. Finally, we sought feedback on the curriculum itself and how satisfied residents were with the education provided. For each outcome measure, we only included subjects with complete data for all questions/measures (e.g., all 56 questions for the knowledge test).

Trainees who subsequently participated in our upper-level POCUS elective were retested with the same assessment tools to assess decay in knowledge.

### Data analysis

We calculated summative statistics, including proportions, medians and interquartile ranges as appropriate. We used paired t tests, two-tailed, to compare pre- and post-curriculum knowledge test scores and confidence with POCUS use, with a p-value < 0.05 considered significant. All calculations were performed using Microsoft Excel™ (Redmond, WA).

## Results

### Curriculum implementation and outcomes

Over the course of 3 years, we taught 87 categorical IM interns in our longitudinal curriculum; 37 (42.5%) trainees completed all pre- and post-knowledge test questions.

### POCUS knowledge

Knowledge assessment scores improved significantly post-curriculum. Pre-curriculum, the average score was 44.4% compared to 62.9% after the curriculum, with a mean difference of 18.5% (95%CI 14.9-22.1, p < 0.001).

### Confidence assessments

Thirty-four interns completed all pre- and post-curriculum confidence assessments. These trainees demonstrated significant improvement in confidence for both simple identification of pathology (Table 1) and clinical integration (Table 2). Post-curriculum, interns reported the highest confidence in identifying ascites (86.5 out of max 100, mean difference 37.9, 95% CI 29.3-46.5) and the lowest confidence in identifying an abdominal aortic aneurysm (AAA) (45.4 out of 100, mean difference 14.2, 95% CI 6.6-21.9). Pre- and post-curriculum confidence in using POCUS for evaluating hypotension, hypoxia or dyspnea, and acute kidney injury increased significantly for all three clinical scenarios (Table 2).

**Table 1.** Trainee Confidence with POCUS Identification Pre- and Post-curriculum (n=34). Trainee confidence (0-100) with POCUS identification. Hydronephrosis identification was moderate to severe, knee effusion was moderate to large. AAA, abdominal aortic aneurysm; CI, confidence interval; DVT, deep venous thrombosis; EF, ejection fraction; IVC, inferior vena cava; POCUS, point of care ultrasound; PTX, pneumothorax.

Clinical Scenario	Pre-curriculum	Post-curriculum	Mean Difference	95% CI	p-value
Pericardial effusion	41.6	71.9	30.2	21.8-38.6	<0.001
Reduced EF	31.7	68.1	36.4	27.2-45.5	<0.001
IVC size & variation	41.6	67.7	26.1	18.3-33.9	<0.001
Pulmonary edema	32.0	69.4	37.5	29.6-45.3	<0.001
Pneumonia	27.4	57.0	29.6	20.0-39.3	<0.001
Pleural effusion	38.9	68.9	30.1	19.7-40.4	<0.001
PTX (rule out)	36.0	66.9	30.9	22.8-39.0	<0.001
Ascites	48.6	86.5	37.9	29.3-46.5	<0.001
Cellulitis	23.6	53.4	29.8	21.6-38.0	<0.001
Abscess	34.7	62.2	27.5	19.9-35.1	<0.001
DVT	30.6	61.9	31.3	22.7-39.9	<0.001
AAA	31.1	45.4	14.2	6.6-21.9	<0.001
Hydronephrosis	32.4	75.0	42.6	33.4-51.8	<0.001
Knee effusion	35.8	57.1	21.4	14.0-28.7	<0.001

**Table 2.** Trainee Confidence in Applying Point of Care Ultrasound (POCUS) to Clinical Scenarios (n=34).

Clinical Scenario	Pre-curriculum	Post-curriculum	Mean Difference	95% CI	p-value
Evaluating cause for hypotension	28.4	66.7	38.4	31.5-45.2	<0.001
Evaluating hypoxia or dyspnea	25.2	64.4	39.2	32.3-46.1	<0.001
Evaluating cause of acute kidney injury	26.1	64.4	38.3	30.8-45.1	<0.001

### POCUS satisfaction and barriers to POCUS use

Thirty-five interns completed all post-curriculum satisfaction questions (Table 3). While 53.2% agreed that the use of POCUS adds significant time to clinical work, 86.1% agreed that the use of POCUS for bedside diagnosis adds significant value, and 79.4% agreed that POCUS increased satisfaction with their clinical work. Most (94.3%) indicated that they were either likely or very likely to continue to use POCUS in clinical practice. Barriers to POCUS use, in order of most to least selected, included lack of time, equipment availability, training, and patient factors.

**Table 3.** Trainee Post-Curriculum Survey Regarding Attitudes to Point of Care Ultrasound (POCUS) (n=35).

Question	(%) Agreement
The use of POCUS for bedside diagnosis adds significant time requirements to my clinical work.	53.2
The use of POCUS for bedside diagnosis adds significant value to my clinical work.	86.1
The use of POCUS increases my confidence in my bedside diagnosis.	79.4
The use of POCUS increases satisfaction with my clinical work.	78.9
How do you assess balance of time versus value for POCUS?	
POCUS adds more time burden than value	0
POCUS adds adequate value for time burden	60.0
POCUS adds more value than time burden	40.0
How likely are you to continue to use POCUS in clinical practice?	
Very likely	51.4
Likely	42.9
Unsure	5.7
Unlikely	0
Very unlikely	0
What are barriers to your use of POCUS in future? (multiple answers permitted)	
Lack of time	88.6
Equipment availability	80.0
Training	34.3
Patient factors	20.0
Other	2.9
Cleaning equipment	0
How often do you anticipate using POCUS after this elective?	
0–5 times/week	54.3
6–10 times/week	37.1
11–15 times/week	8.6
16–20 times/week	0
>20 times/week	0

### POCUS curriculum satisfaction

Overall, interns strongly agreed that they were satisfied with the curriculum (mean 91.2 out of 100, SD 9.7) and that it provided the skill set needed to successfully perform POCUS examinations (mean 84.2 out of 100, SD 13.3). Suggestions to improve the curriculum included having even more sessions and more opportunities for clinical integration.

### Knowledge decay

Of the 37 interns included in our assessment, there were 15 who subsequently participated in our 2-week upper-level POCUS elective and completed a repeat pre-elective knowledge test (40.5%). The median time between assessments was 14 months (IQR 6.25, 17). There was no statistically significant difference between the knowledge assessments of the 15 residents who completed the intern curriculum and then opted to take the upper-level elective (mean intern post-test score was 68.6% with mean elective pre-test 66.3%; mean difference 2.3%, 95%CI -3.4-8.0, p = 0.41).

## Discussion

In this study of the first three years of experience following the implementation of a longitudinal POCUS curriculum for IM interns, trainees demonstrated significant gains in POCUS knowledge. Post-curriculum, participants also reported improved confidence in their ability to use POCUS to identify specific pathologies and apply this to clinical scenarios. Among those who subsequently participated in the intensive POCUS elective as upper-level residents, there was no decrease in knowledge comparing post-intern curriculum to pre-elective knowledge tests, despite a median 14-month gap between assessments. This suggested good knowledge retention. Lastly, interns reported high levels of satisfaction with the curriculum and planned to incorporate POCUS in their future clinical practice. Our curriculum structure, with sessions spaced every six weeks through the academic year, may be more feasible than shorter, intensive curricula for Accreditation Council for Graduate Medical Education (ACGME) programs to incorporate within standardized training requirements and thus may be able to serve as a model for other programs.

For knowledge acquisition and retention to be replicated in future POCUS or medical education curricula, we posit a few mechanisms for our success. First, the longitudinal structure allowed for knowledge application and had been shown to be superior to single-session or short-term POCUS curricula [18,19]. Second, the spaced teaching sessions, which presented new concepts each time while also reinforcing key concepts, provided opportunities for informal repetition of fundamental material. Third, the curriculum structure provided tools for autonomous learning outside of the dedicated curriculum time, which is a fundamental tenet of adult learning theory and may have contributed to increased confidence in POCUS knowledge after the curriculum [23]. Importantly, the intern longitudinal curriculum was only a part of our Basal-Bolus POCUS curriculum. Between sessions, interns rotated through the medicine procedure service and intensive care units which served as “boluses” of opportunities to engage in deliberate practice of newly acquired skills under the guidance of POCUS and procedural faculty in real-world clinical scenarios.

Several prior POCUS curricula for IM trainees have been described [15–19]. While a number of prior curricula were also longitudinal, some were elective throughout or part of the curriculum implementation rather than being required for all trainees [15,17–19]. Additionally, two of the longitudinal curricula were implemented over a six-month time frame rather than the whole academic year [18,19]. The study most similar to ours was by Faiella et al., though the curriculum was implemented in a Canadian academic center [17]. The Canadian curriculum also included training for all interns, with spaced sessions through the academic year. However, they implemented 30 shorter sessions more frequently, compared with our material. The majority of the studies reported results of pre- and post-curricular knowledge tests [15–17,19]. It should be noted that, as of yet, there is not one accepted standard for post-curriculum POCUS knowledge, which is reflected by the fact that the four studies above each created their own test, ranging from 11 to 30 questions in length. The mean post-test score range in prior studies (70.2–82.8%) was higher than our post-curriculum mean of 62.9% [15–17,19]. This is likely due in part to the difficulty of our 56-question assessment, consciously aligned with Society of Hospital Medicine (SHM) and American College of Chest Physicians (CHEST) POCUS certification standards, which are more stringent than in previous studies.

Limitations of our study include relatively low response rates for post-curriculum assessments of knowledge, confidence, and retention. Non-responders may differ from those who participated in subsequent assessments. We plan to address this by continuing the curriculum to increase the sample size of completed assessments. Adding dedicated in-person time at the beginning and end of the curriculum for the residents to take the knowledge test could improve response rates. Additionally, the assessment of knowledge retention was conducted among upper-level residents who took the POCUS elective and thus may have more baseline interest and knowledge of POCUS compared with trainees who did not opt to participate in this elective. We also did not assess skill acquisition and retention for the intern longitudinal curriculum; although, hands-on skills testing is an important part of the upper-level POCUS elective. Haptic competency will be a necessary component for developing a POCUS curriculum if IM follows emergency and family medicine in making POCUS a required graduate medical education competency. Relatedly, we should note that while important, the confidence in use of POCUS that we measured does not necessarily correlate with competence. Lastly, our study was conducted in a large university setting with multiple faculty members trained in POCUS and thus may not be generalizable to other training centers, particularly given the capital equipment and number of POCUS-competent faculty required to successfully implement a similar program.

## Conclusions

Our year-long intern longitudinal POCUS curriculum led to improved knowledge and confidence in POCUS applications and was well received by trainees, though hands-on competency was not directly assessed. Additionally, our data revealed minimal decay in knowledge and confidence between the completion of the intern curriculum and the beginning of the more intensive upper-level elective. We propose this basal component as the foundation of a successful residency-long POCUS curriculum that can serve as a framework for other programs seeking to incorporate POCUS training. At our institution, POCUS education throughout 3 years of residency includes bolus components (such as our upper-level elective) in addition to the basal component described in this study. Continued education after the intern year is essential to reinforce skills, and it is important that formal teaching does not cease after completion of the intern curriculum. We intend to describe the second half of our basal-bolus curriculum in future publications. Other areas for future study could include assessment of hands-on competency or measurement of patient care metrics, such as length of stay or earlier detection of patient decompensation for providers before and after completion of POCUS training. Finally, as POCUS education expands among IM residencies in different formats, defining “competency” will need to be addressed further to provide clear benchmarks for graduating physicians.
