# Supplementary material for: Laying a Strong Foundation with a Novel “Basal-Bolus” Point of Care Ultrasound Curriculum for Internal Medicine Residents
Source: POCUS J. 2026 Apr 22;11(1):15–21. doi: 10.24908/pocusj.v11i01.20051 (PMC13161781; doi:10.24908/pocusj.v11i01.20051)
Supplement: Supplementary file 1 [file pocusj-11-01-20051-s001.pdf]

Supplementary Material S1

Pre- and post-knowledge assessments:
We are unable to publish the entire knowledge exam because some of the images have been published and copyrighted in other journals since we created the test. We have included the learning objectives of each of our 56 questions to the knowledge test and a subset of representative questions with original images that have not been previously published.

Learning objectives:

1. Apply the most appropriate ultrasound settings to maximize resolution of cardiac imaging.
2. Recognize posterior shadowing and correctly interpret this artifact to identify a bladder stone.
3. Recognize reverberation artifact as a characteristic of a central line guidewire.
4. Recognize that using POCUS during a code should not increase the pulse check time.
5. Identify normal sonoanatomy of the coronal right upper quadrant view and exclude pleural effusion, abdominal free fluid, and hydronephrosis.
6. Recognize severe hydronephrosis.
7. Differentiate anatomy above and below the diaphragm and identify a full stomach on coronal left upper quadrant view of the abdomen.
8. Differentiate normal peristalsis from to-and-fro movement in the small bowel.
9. Describe the probe movements necessary to visualize the kidney.
10. Differentiate hydronephrosis and polycystic kidney disease.
11. Recognize the spine sign and interpret it correctly as a sign of pathology above the diaphragm in a standard right upper quadrant coronal view.
12. Identify lung point and recognize its accuracy in diagnosing pneumothorax.
13. Recognize a developing abscess and formulate a management plan for skin and soft tissue infections.
14. Recognize a popliteal vein thrombus and differentiate the site of thrombosis from other levels of the femoral vein as well as common mimics.
15. Identify dynamic air bronchograms and their clinical significance and develop an appropriate management plan for lobar pneumonia.
16. Interpret left ventricular function in the parasternal long and short axis views to differentiate causes of shock.
17. Interpret a 1cm hypoechoic, pleural based, wedge-shaped lesion as a potential sign of lung cancer or pulmonary embolism.
18. Interpret B-lines as interstitial syndrome, differentiate diagnoses that can cause B-lines using the patient's history, and formulate a management plan for patients with heart failure and B-lines suggestive of pulmonary edema.
19. Estimate right atrial pressure using inferior vena cava (IVC) size and collapsibility.
20. Distinguish simple vs complex pleural effusions and formulate a management plan for a loculated pleural effusion.
21. Recognize that the IVC does not measure "volume status." It is a measure of pressure and can be influenced by increased intrathoracic pressure.
22. Identify the structures in a parasternal long axis view of the heart.
23. Describe the steps to calculate a bladder volume using POCUS.

24. Identity the greater saphenous vein intake of the common femoral vein and interpret the significance of full compressibility with regard to absence of thrombus.
25. Recognize the falciform ligament on the subcostal 4 chamber view of the heart and interpret its presence as a sign of abdominal ascites.
26. Identify the chambers on a standard apical 4 chamber view of the heart.
27. Demonstrate correct measurement of the spleen and identify splenomegaly.
28. Identify hydronephrosis and recognize that if a stone is not clearly visualized, more information is needed to determine the cause.
29. Identify pelvic free fluid in the rectovaginal/rectouterine space in a female in a transverse view.
30. Identify the uterus in a sagittal view of the female pelvis.
31. Identify the seminal vesicles in a male on a transverse view of the bladder and distinguish them from free fluid.
32. Distinguish an obstructed indwelling catheter in the bladder from free fluid, pathological bowel loops, pregnancy, or bladder malignancy.
33. Identify bilateral hydronephrosis and distended bladder in a patient with acute obstructive nephropathy and then formulate a management plan to relieve the obstruction.
34. Recognize that lack of lung sliding has multiple causes in addition to pneumothorax.
35. Identify B-lines.
36. Identify consolidated lung due to a large pleural effusion.
37. Identify the confluence of the deep femoral and superficial femoral veins to become the common femoral vein.
38. Identify the confluence of the greater saphenous vein and the common femoral vein.
39. Distinguish the superficial femoral vein from the superficial femoral artery when neither vessel is compressible due to a thrombus in the vein.
40. Recognize the significance of complete compression during an extended compression DVT exam and differentiate a Baker's cyst from vascular pathology.
41. Evaluate the accuracy of estimating left ventricular function at different levels of the parasternal short axis view.
42. Recognize an off-axis longitudinal view of the IVC and infer its lack of utility for volume responsiveness.
43. Recognize a catheter in a longitudinal IVC view.
44. Differentiate an epicardial fat pad on the subcostal 4 chamber view from a pericardial effusion.
45. When cardiac views are limited by poor windows, recognize that a single parasternal long axis view is insufficient to rule out a pericardial effusion or right ventricular pathology, or to determine fluid responsiveness.
46. Estimate the ejection fraction of a poorly contracting heart as > or < 40% on a standard parasternal long axis view.
47. Identify a pericardial effusion and assess the likelihood of tamponade based on findings such as right atrial and ventricular collapse and the size and collapsibility of the IVC.
48. Identify a pericardial effusion and assess the likelihood of tamponade based on the size and collapsibility of the IVC.
49. Identify the chambers on an off-center apical 4 chamber view and recognize a severely dilated right atrium.

50. Predict which of the views of the heart (parasternal long axis, parasternal short axis, apical 4 chamber, and subcostal 4 chamber) would be the least reliable for detecting increased right ventricular chamber size because it gives the most limited view of the right ventricle.
51. Recognize that valvular pathology, especially the question of endocarditis, is outside the scope of basic POCUS and requires cardiology input.
52. Identify the celiac trunk and superior mesenteric artery branches of the aorta in a transverse view.
53. Correctly measure the outer wall to outer wall diameter of an abdominal aortic aneurysm.
54. Identify an aortic dissection in transverse orientation.
55. Differentiate a lymph node from an abscess using color Doppler and formulate a management plan for lymphadenopathy.
56. Identify a suprapatellar knee effusion in the longitudinal orientation

Representative Questions:

1) 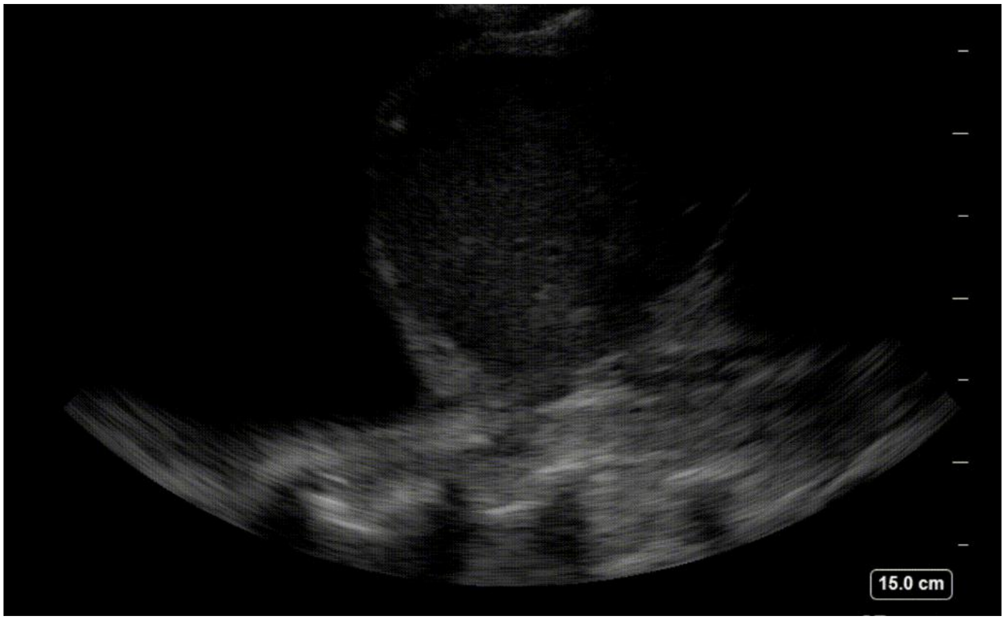

   a. Question: This video clip, taken from the right posterior axillary line, demonstrates:
   b. Answer choices:
      i. Free peritoneal fluid
      ii. Simple pleural effusion
      iii. Complicated pleural effusion
      iv. Hydronephrosis
      v. Liver cysts
   c. Learning objective: Recognize the spine sign and interpret it correctly as a sign of pathology above the diaphragm in a standard right upper quadrant coronal view.

2) 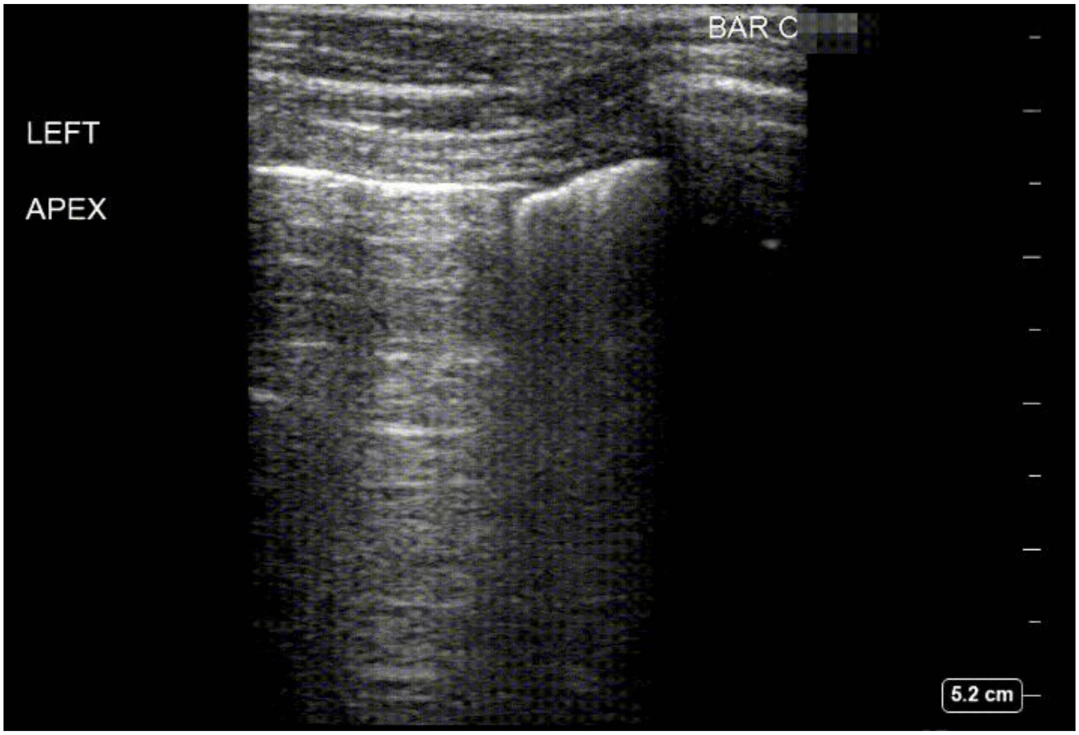

a. Question: this lung ultrasound finding is specific for:
b. Answer choices:
    i. Normal lung
    ii. Pneumonia
    iii. Pneumothorax
    iv. Pulmonary fibrosis
    v. Pulmonary edema
c. Learning objective: Identify lung point and recognize its accuracy in diagnosing pneumothorax.

3)

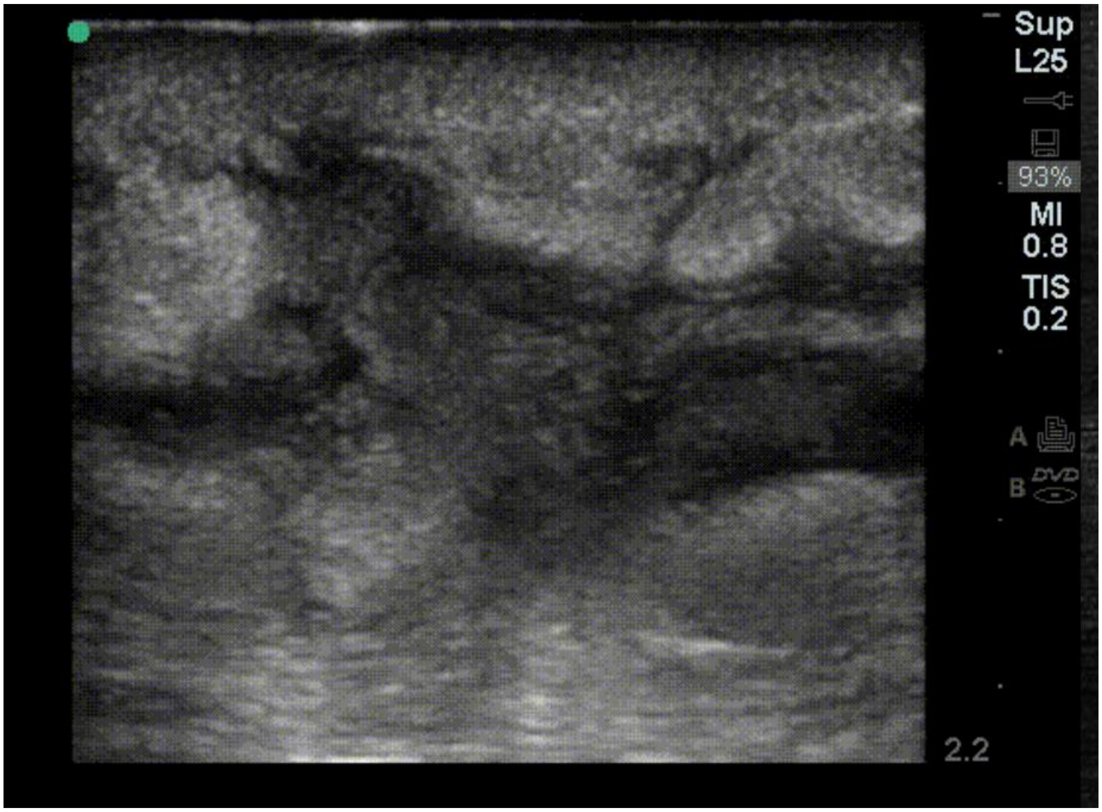

a. Question: This 46-year-old patient with morbid obesity, diabetes, and peripheral vascular disease presents with pain, swelling, and redness in the lower extremity. He has a temp of 38.1 C, but his vitals are otherwise normal, and he is not in distress. Lower extremity ultrasound is shown. What is the most appropriate next step in the management of this patient?
b. Answer choices:
    i. Initiation of antibiotics alone
    ii. Incision and drainage of the fluid collection and initiation of antibiotics
    iii. STAT surgery consult for fasciotomy
    iv. Apply color Doppler for better characterization of the lesion
    v. Start therapeutic anticoagulation for DVT
c. Learning objective: Recognize a developing abscess and formulate a management plan for skin and soft tissue infections

4) 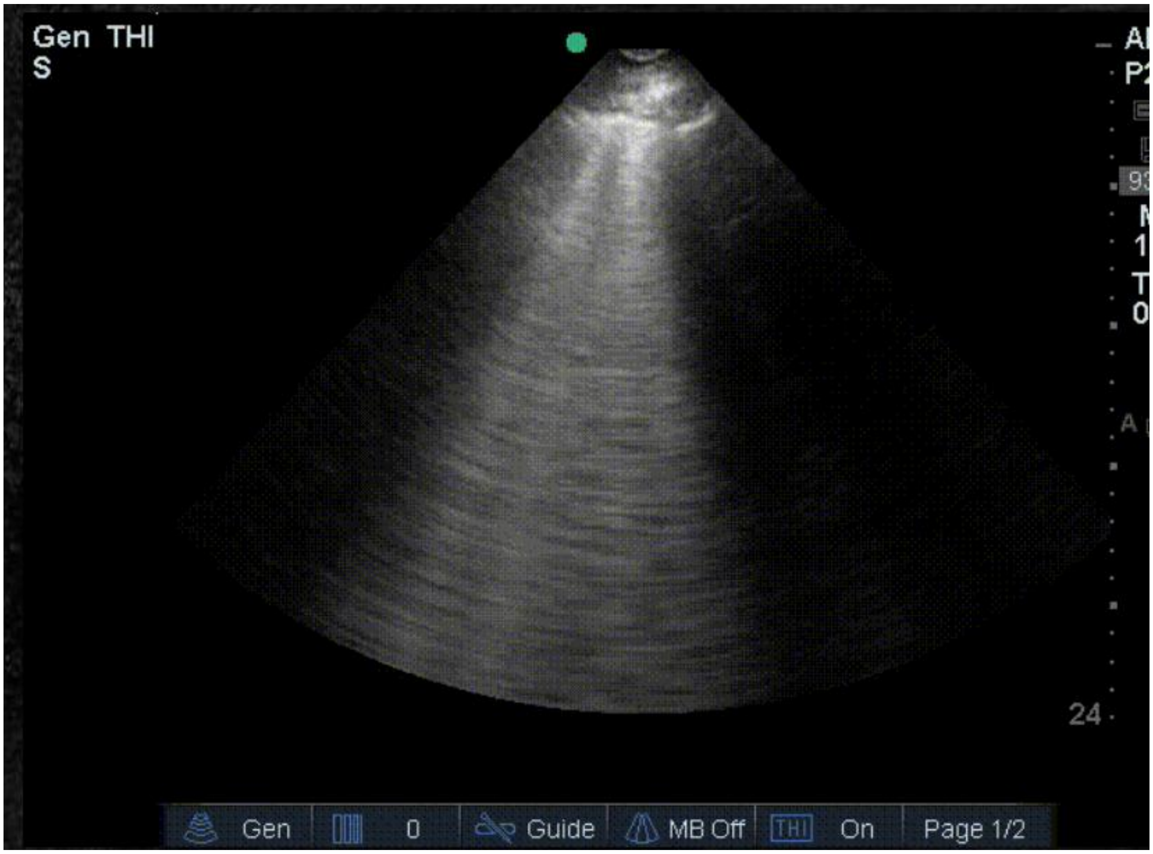

a. Question: A 54-year-old woman with past medical history of diastolic heart failure (HFPEF) with her last ejection fraction documented at 65%, hypertension, and COPD, presents with 2 days of dyspnea. She reports new cough and increased wheezing. She is afebrile. Heart rate is 105 bpm, blood pressure 105/70 mmHg, respiratory rate 22 breaths/minute, pulse oximetry 83% on room air. An ultrasound video clip, representative of findings in multiple zones bilaterally, is provided below. Based on the ultrasonographic findings, this patient will most benefit from:
b. Answer choices:
   i. Antibiotics
   ii. Beta agonists
   iii. Unfractionated heparin
   iv. Loop diuretics
   v. Thoracentesis

5) Learning objective: Interpret B-lines as interstitial syndrome, differentiate diagnoses that can cause B-lines using the patient's history, and formulate a management plan for patients with heart failure and B-lines suggestive of pulmonary edema.

6) 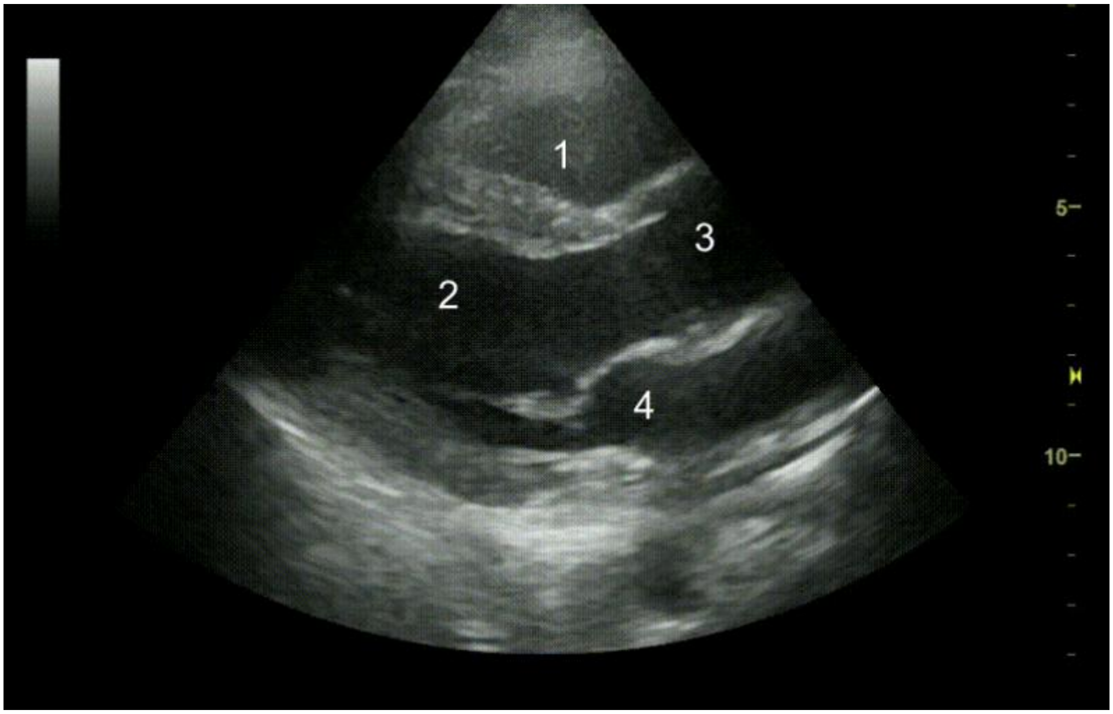

a. Question: This is a recording from a 66-year-old man with a history of hypertension who presented with shortness of breath. Which one of the labeled structures is right atrium?
b. Answer choices:
    i. 1
    ii. 2
    iii. 3
    iv. 4
    v. None of the above
c. Learning objective: Identify the structures in a parasternal long axis view of the heart.

7) 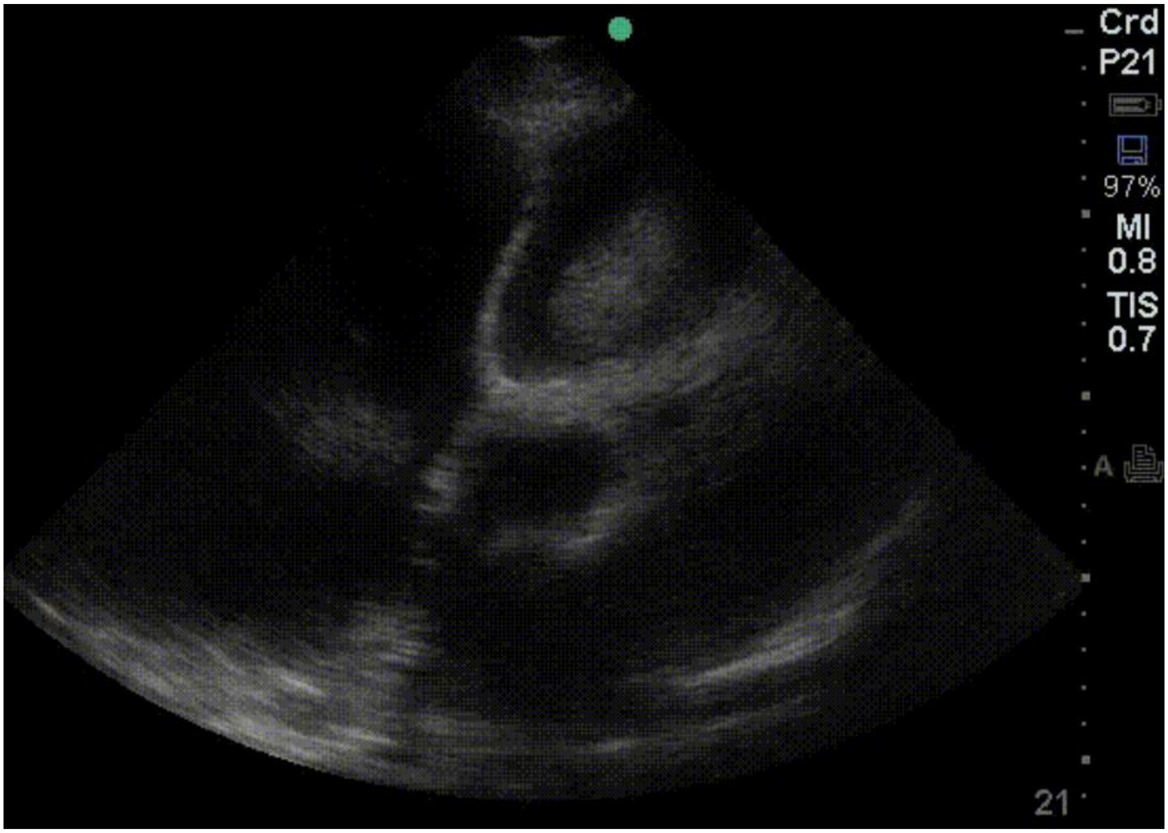

a. Question: In this subcostal view of the heart:
b. Answer choices:
   i. **Ascites is present**
   ii. The right ventricle is larger than the left ventricle
   iii. A left sided pleural effusion is seen
   iv. There is both ascites and pleural effusion present
   v. The left ventricle is hyperdynamic
c. Learning objective: Recognize the falciform ligament on the subcostal 4 chamber view of the heart and interpret its presence as a sign of abdominal ascites.

8) 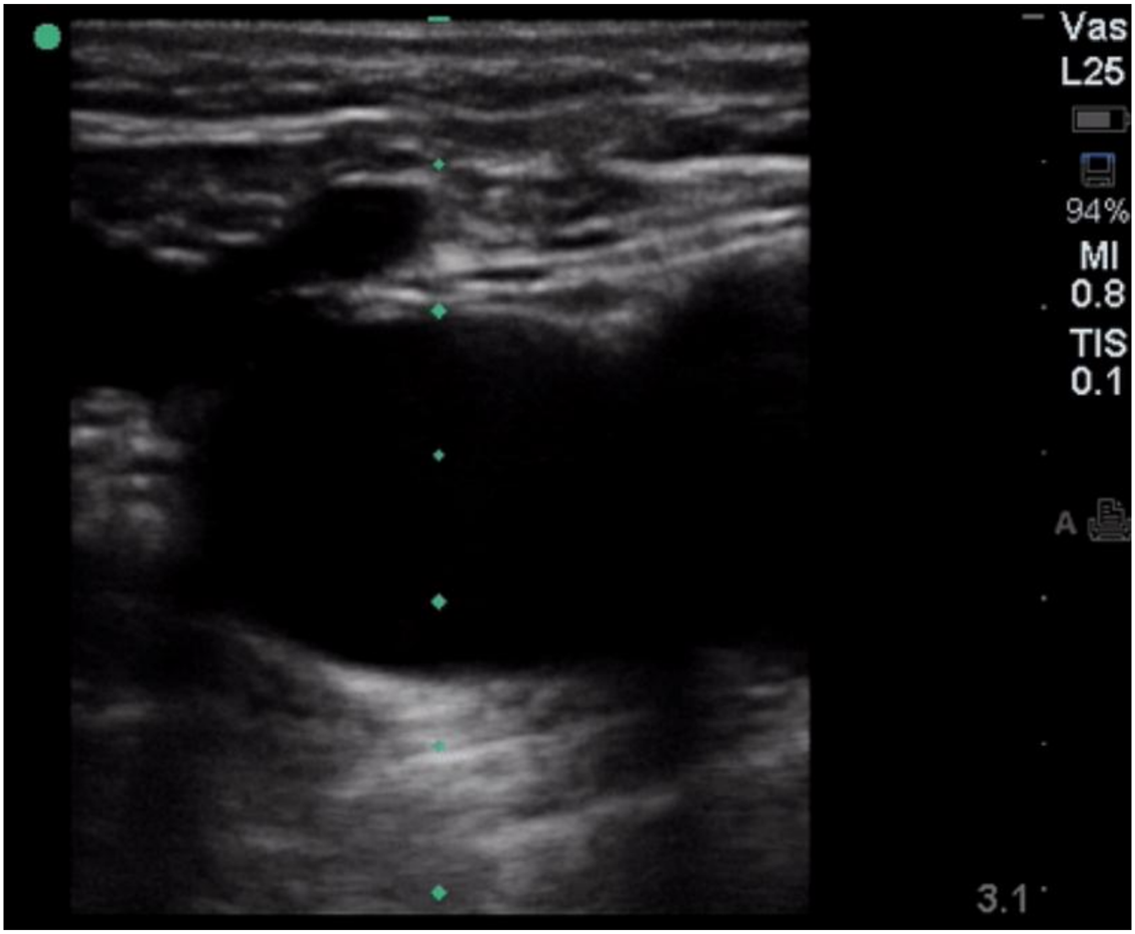

a. Question: Identify the level of this left leg DVT study.
b. Answer choices:
    i. Common femoral vein
    ii. Saphenous vein confluence
    iii. Deep femoral vein confluence
    iv. “Superficial” femoral vein
    v. Popliteal vein
c. Learning objective: Identify the confluence of the greater saphenous vein and the common femoral vein.

9) 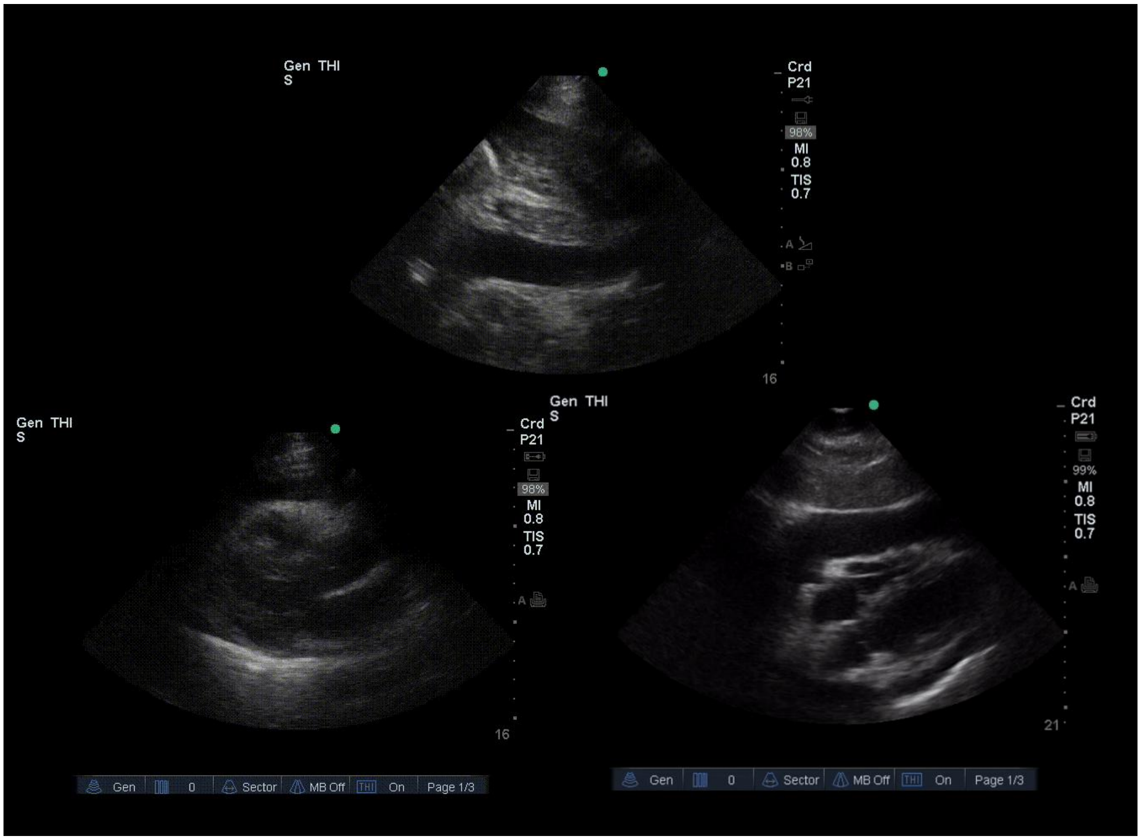

a. Question: Which of the following statements is present in the following 3 clips?
b. Answer choices:
    i. Large pleural effusion
    ii. Large pericardial effusion WITHOUT suggestion of tamponade
    iii. Large pericardial effusion, WITH suggestion of tamponade
    iv. Severely reduced EF
    v. Normal post-op changes after pericardial window
c. Learning objective: Identify a pericardial effusion and assess the likelihood of tamponade based on the size and collapsibility of the IVC.

10) No image

a. Question: While evaluating several different views of your patient's heart, you notice that the RV seems larger in some views than others. Which view is least reliable for detecting increased RV size?

b. Answer choices:

   i. **Parasternal long axis (PLAX)**

   ii. Parasternal short axis (PSAX)

   iii. Apical 4 chamber

   iv. Subcostal 4 chamber

c. Learning objective: Predict which of the views of the heart (parasternal long axis, parasternal short axis, apical 4 chamber, and subcostal 4 chamber) would be the least reliable for detecting increased right ventricular chamber size because it gives the most limited view of the right ventricle.

11) 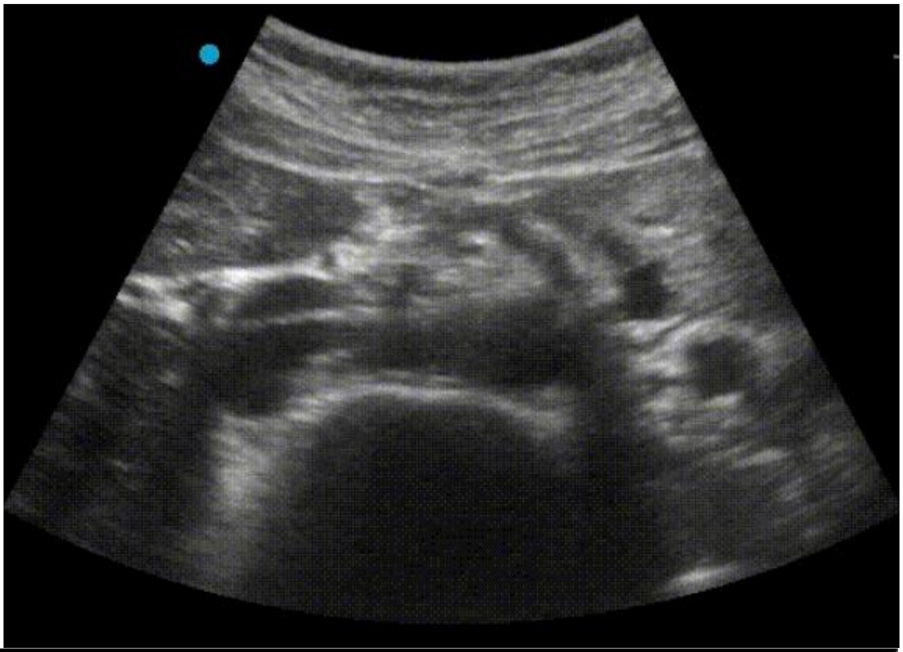

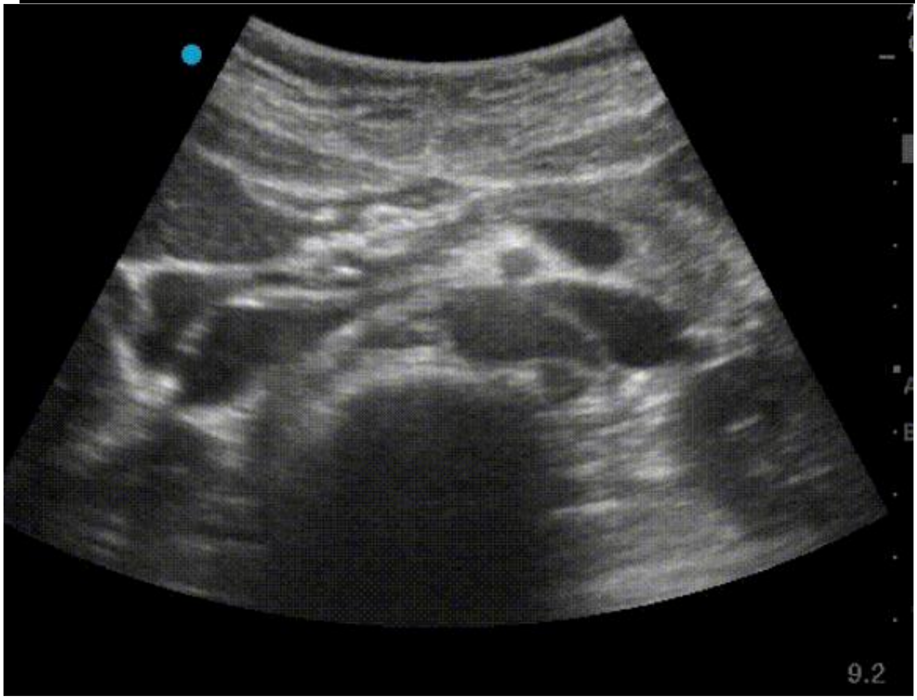

a. Question: The following clip shows:
b. Answer choices:
    i. The IVC with hepatic vein confluence
    ii. The IVC with renal veins
    iii. The abdominal aorta with celiac trunk and SMA
    iv. The abdominal aorta at the iliac bifurcation
    v. Liver with normal gallbladder
c. Learning objective: Identify the celiac trunk and superior mesenteric artery branches of the aorta in a transverse view.

12) 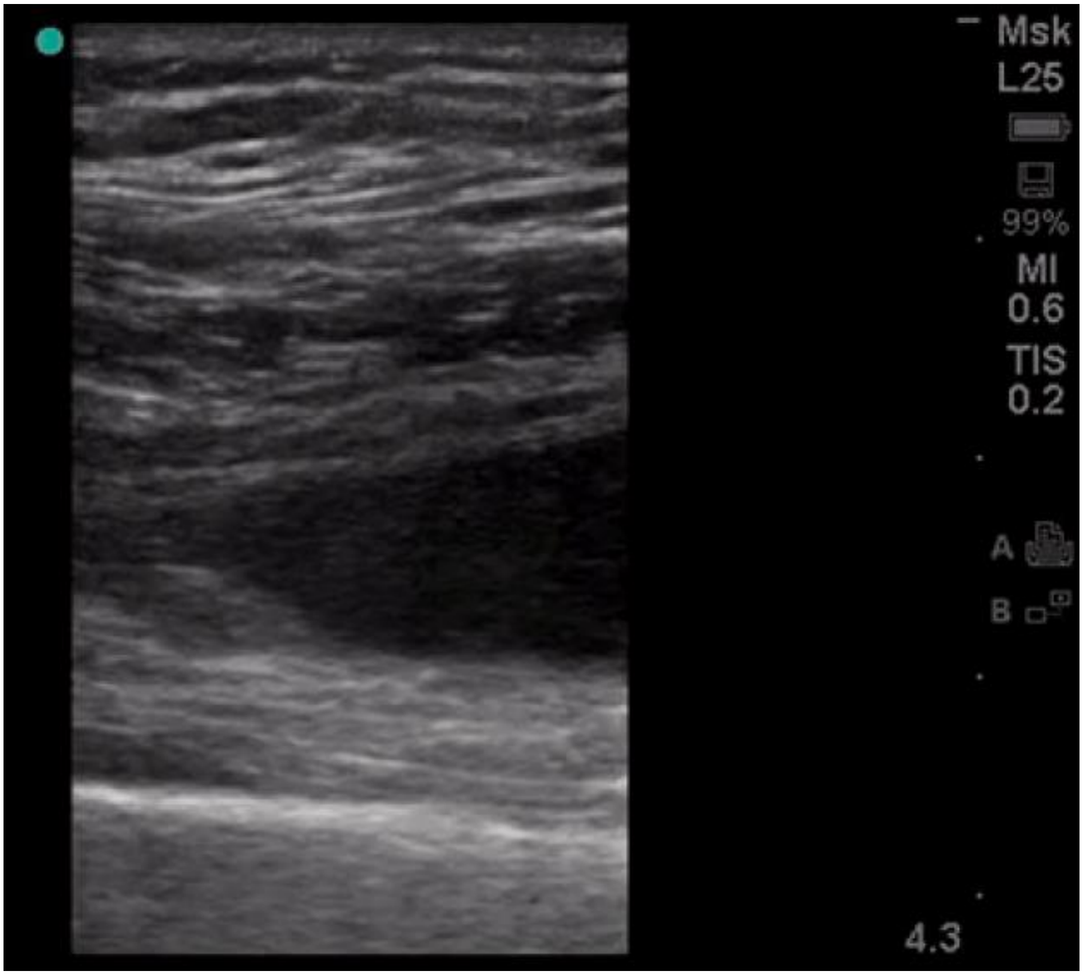

a. Question: Patient presents with pain, swelling, and redness over the left knee. Ultrasound of the knee joint demonstrates which of the following?
b. Answer choices:
    i. Normal anatomy
    ii. Tendon rupture
    iii. Effusion
    iv. Hemarthrosis
    v. Cutaneous abscess
c. Learning objective: Identify a suprapatellar knee effusion in the longitudinal orientation
