# Supplementary material for: Laying a Strong Foundation with a Novel “Basal-Bolus” Point of Care Ultrasound Curriculum for Internal Medicine Residents
Source: POCUS J. 2026 Apr 22;11(1):15–21. doi: 10.24908/pocusj.v11i01.20051 (PMC13161781; doi:10.24908/pocusj.v11i01.20051)
Supplement: Supplementary file 2 [file pocusj-11-01-20051-s002.pdf]

Supplementary Material S2

Confidence assessment

**POCUS Skills**	
Did you receive any point of care ultrasound (POCUS) training in medical school	◯ Yes<br>◯ No
How many years of POCUS training did you have in medical school?	◯ 1<br>◯ 2<br>◯ 3<br>◯ 4
Did you receive POCUS education during anatomy teaching?	◯ Yes<br>◯ No
Did you receive POCUS education during your clinical years?	◯ Yes<br>◯ No
Have you ever been trained in POCUS outside of medical school?	◯ Yes<br>◯ No
Did your school have formal POCUS education? If so, please provide any details if possible	____________________
How confident are you in your ability to identify a pericardial effusion using POCUS?	Not at all Confident — Moderately Confident — Extremely Confident<br>*(Place a mark on the scale above)*
How confident are you in your ability to identify moderate-severely decreased EF (< 40%) using POCUS?	Not at all Confident — Moderately Confident — Extremely Confident<br>*(Place a mark on the scale above)*
How confident are you in your ability to identify IVC size and variation using POCUS?	Not at all Confident — Moderately Confident — Extremely Confident<br>*(Place a mark on the scale above)*
How confident are you in your ability to identify lung interstitial fluid using POCUS?	Not at all Confident — Moderately Confident — Extremely Confident<br>*(Place a mark on the scale above)*
How confident are you in your ability to identify lung consolidation using POCUS?	Not at all Confident — Moderately Confident — Extremely Confident<br>*(Place a mark on the scale above)*
How confident are you in your ability to identify a pleural effusion using POCUS?	Not at all Confident — Moderately Confident — Extremely Confident<br>*(Place a mark on the scale above)*

How confident are you in your ability to rule out a pneumothorax using POCUS?	Not at all Confident	Moderately Confident	Extremely Confident
	(Place a mark on the scale above)		
How confident are you in your ability to identify ascites using POCUS?	Not at all Confident	Moderately Confident	Extremely Confident
	(Place a mark on the scale above)		
How confident are you in your ability to identify cellulitis using POCUS?	Not at all Confident	Moderately Confident	Extremely Confident
	(Place a mark on the scale above)		
How confident are you in your ability to identify an abscess using POCUS?	Not at all Confident	Moderately Confident	Extremely Confident
	(Place a mark on the scale above)		
How confident are you in your ability to identify a lower extremity deep venous thrombosis using POCUS?	Not at all Confident	Moderately Confident	Extremely Confident
	(Place a mark on the scale above)		
How confident are you in your ability to identify an abdominal aortic aneurysm using POCUS?	Not at all Confident	Moderately Confident	Extremely Confident
	(Place a mark on the scale above)		
How confident are you in your ability to identify moderate to severe hydronephrosis using POCUS?	Not at all Confident	Moderately Confident	Extremely Confident
	(Place a mark on the scale above)		
How confident are you in your ability to identify a moderate to large knee effusion using POCUS?	Not at all Confident	Moderately Confident	Extremely Confident
	(Place a mark on the scale above)		

POCUS Integration

How confident are you in your ability to use POCUS to evaluate the cause of hypotension?	Not at all Confident	Moderately Confident	Extremely Confident
	(Place a mark on the scale above)		
How confident are you in your ability to use POCUS to evaluate the cause of hypoxia or dyspnea?	Not at all Confident	Moderately Confident	Extremely Confident
	(Place a mark on the scale above)		
How confident are you in your ability to use POCUS to evaluate the cause of an AKI?	Not at all Confident	Moderately Confident	Extremely Confident
	(Place a mark on the scale above)		

POCUS Education Quality

I am Satisfied with the curriculum of the POCUS elective	Strongly Disagree — Neither agree or disagree — Strongly Agree<br>*(Place a mark on the scale above)*
The POCUS elective has given me the skill set needed to successfully perform ultrasound examinations.	Strongly Disagree — Neither agree or disagree — Strongly Agree<br>*(Place a mark on the scale above)*
What feedback would you like to provide on the POCUS curriculum?	______________________________
