# Supplementary material for: Laying a Strong Foundation with a Novel “Basal-Bolus” Point of Care Ultrasound Curriculum for Internal Medicine Residents
Source: POCUS J. 2026 Apr 22;11(1):15–21. doi: 10.24908/pocusj.v11i01.20051 (PMC13161781; doi:10.24908/pocusj.v11i01.20051)
Supplement: Supplementary file 3 [file pocusj-11-01-20051-s003.pdf]

Supplementary Material S3

POCUS and curriculum satisfaction

**POCUS Satisfaction**	
The use of POCUS for bedside diagnosis adds significant time requirements to my clinical work.	Strongly Disagree — Neither agree or disagree — Strongly Agree<br>*(Place a mark on the scale above)*
The use of POCUS for bedside diagnosis adds significant value to my clinical work.	Strongly Disagree — Neither agree or disagree — Strongly Agree<br>*(Place a mark on the scale above)*
With which of the following statements regarding the balance of burden and value of POCUS do you most agree?	◯ Using POCUS adds more time burden than value to my clinical work.<br>◯ Using POCUS adds enough value to my clinical work to justify the time burden.<br>◯ Using POCUS adds more value than time burden to my clinical work.
The use of POCUS increases my confidence in my bedside diagnosis.	Strongly Disagree — Neither agree or disagree — Strongly Agree<br>*(Place a mark on the scale above)*
How does the use of POCUS affect your satisfaction with your clinical work?	Strongly Disagree — Neither agree or disagree — Strongly Agree<br>*(Place a mark on the scale above)*
How many times a week do you anticipate having the opportunity to use (if taking this survey at the end of your elective)/ have you had the opportunity to use (if taking this survey 6 months post-elective) POCUS in direct patient care?	◯ 0-5<br>◯ 6-10<br>◯ 11-15<br>◯ 16-20<br>◯ >21
What are barriers to the use of POCUS in your clinical work? (select all that apply)	☐ Equipment Availability<br>☐ Lack of Time<br>☐ Patient Factors<br>☐ Cleaning Equipment<br>☐ Training<br>☐ Other
Other Barriers:	______________________
How likely are you to continue to use POCUS in your clinical practice?	◯ Very Unlikely<br>◯ Unlikely<br>◯ Unsure<br>◯ Likely<br>◯ Very Likely

POCUS Education Quality

I am Satisfied with the curriculum of the POCUS elective	Strongly Disagree — Neither agree or disagree — Strongly Agree<br>*(Place a mark on the scale above)*
The POCUS elective has given me the skill set needed to successfully perform ultrasound examinations.	Strongly Disagree — Neither agree or disagree — Strongly Agree<br>*(Place a mark on the scale above)*
What feedback would you like to provide on the POCUS curriculum?	________________________________
